# Cardiomyocyte Antihypertrophic Effect of Adipose Tissue Conditioned Medium from Rats and Its Abrogation by Obesity is Mediated by the Leptin to Adiponectin Ratio

**DOI:** 10.1371/journal.pone.0145992

**Published:** 2016-01-05

**Authors:** Suresh C. Bairwa, Venkatesh Rajapurohitam, Xiaohong Tracey Gan, Rabban Mangat, Spencer D. Proctor, Morris Karmazyn

**Affiliations:** 1 Department of Physiology and Pharmacology, Schulich School of Medicine and Dentistry, University of Western Ontario, London, Ontario, Canada N6A 5C1; 2 Alberta Diabetes and Mazankowski Heart Institutes, University of Alberta, Edmonton, Alberta, Canada T6G 2E1; Graduate School of Medicine, Osaka University, JAPAN

## Abstract

White adipocytes are known to function as endocrine organs by secreting a plethora of bioactive adipokines which can regulate cardiac function including the development of hypertrophy. We determined whether adipose tissue conditioned medium (ATCM) generated from the epididymal regions of normal rats can affect the hypertrophic response of cultured rat ventricular myocytes to endothelin-1 (ET-1) administration. Myocytes were treated with ET-1 (10 nM) for 24 hours in the absence or presence of increasing ATCM concentrations. ATCM supressed the hypertrophic response to ET-1 in a concentration-dependent manner, an effect enhanced by the leptin receptor antagonist and attenuated by an antibody against the adiponectin AdipoR1 receptor. Antihypertrophic effects were also observed with ATCM generated from perirenal-derived adipose tissue. However, this effect was absent in ATCM from adipose tissue harvested from corpulent JCR:LA-cp rats. Detailed analyses of adipokine content in ATCM from normal and corpulent rats revealed no differences in the majority of products assayed, although a significant increase in leptin concentrations concomitant with decreased adiponectin levels was observed, resulting in a 11 fold increase in the leptin to adiponectin ratio in ATCM from JCR:LA-cp. The antihypertrophic effect of ATCM was associated with increased phosphorylation of AMP-activated protein kinase (AMPK), an effect abrogated by the AdipoR1 antibody. Moreover, the antihypertrophic effect of ATCM was mimicked by an AMPK activator. There was no effect of ET-1 on mitogen-activated protein kinase (MAPK) activities 24 hour after its addition either in the presence or absence of ATCM. Our study suggests that adipose tissue from healthy subjects exerts antihypertrophic effects via an adiponectin–dependent pathway which is impaired in obesity, most likely due to adipocyte remodelling resulting in enhanced leptin and reduced adiponectin levels.

## Introduction

It is now well-recognized that adipose tissue has definitive endocrine functionality with the ability to produce and release a large number of bioactive compounds into the circulation [1–3]. Of the known adipocyte-derived compounds several have targets within the cardiovascular system including the heart which can modulate cardiovascular health [4, 5]. The nature of the influence of adipocyte-derived bioactive products including adipokines is difficult to assess since many of these factors possess distinct, and in some cases, opposite properties. Two adipokines which are likely of particular importance to cardiovascular function, leptin and adiponectin, exert opposite effects on the heart and their plasma concentrations are changed in diametrically different directions under cardiovascular disease states [6–8]. In this regard, various studies have shown that plasma leptin levels are elevated whereas adiponectin levels are decreased in patients with cardiovascular disease [9–12]. Interestingly, there are also findings which show similar patterns of changes in plasma leptin and adiponectin concentrations in obesity despite the increase in adiposity, suggesting that decreased adiponectin synthesis or secretion by adipocytes occurs in obese states [13–16].

Adipocytes have been shown to directly supress cardiomyocyte contraction under acute conditions [17, 18] and adipse, little is known as to whether they can modulate more chronic cardiac responses. We therefore hypothesized that adipocyte-derived products present in conditioned medium will modulate the cardiomyocyte response to hypertrophic stimuli. The present study was carried out to address this question, to identify the nature of this modulation and to assess possible underlying mechanisms. Furthermore, we set out to determine whether adipose tissue derived from obese animals possesses altered modulatory properties in their ability to influence the hypertrophic response of cardiomyocytes.

## Materials and Methods

### Cardiomyocyte isolation

The protocol for this study was approved by the Animal Use Subcommittee of the University of Western Ontario (Protocol # 2013–031) and procedures adhered to the guidelines of the Canadian Council on Animal Care (Ottawa, Ontario, Canada). Hearts were extracted from 1–5 day old Sprague–Dawley rats (Charles River Canada, St Constant, Quebec, Canada). After sacrifice by decapitation hearts were excised and rinsed in buffer containing 1X Hank’s balanced salt solution (HBSS) (Wisent Inc., St. Bruno, Quebec, Canada). Hearts were squeezed gently to remove residual blood and transferred to another dish containing fresh ice-cold 1X HBSS. Atrias were removed and ventricles were placed into another dish. Ventricles were minced using a surgical blade and the pieces were transferred to a water-jacketed Erlenmeyer flask maintained at 37°C and digested with digestion buffer containing 10% 10X HBSS, 2% 1 M HEPES, 2% penicillin/streptomycin, 0.11 mg/ml collagenase, 0.13 mg/ml Trypsin and 0.03mg/ml DNase II. After each digestion the supernatant was removed from the flask and poured into an Eppendorf tube having the same volume of stop buffer which consisted of 10% 10 x HBSS, 2% Penicillin/streptomycin and 20% Fetal Bovine Serum (FBS) to abolish collagenase activity. After 6 extractions the solution containing both the cell suspension and stop buffer was filtered using a 70 μm cell strainer and centrifuged at 514 x g at 4°C for 5 minutes. The supernatant was discarded and the cell pellet was re-suspended in culture medium containing Dulbecco’s modified Eagle medium/Ham’s F-12 supplemented with 10% fetal bovine serum, 10 μg/ml transferrin, 2 μg/ml insulin, 10 ng/ml selenium, 50 units/ml penicillin, 50 μg/ml streptomycin, 2 mg/ml bovine serum albumin, 5 μg/ml linoleic acid, 3 mM pyruvic acid, 0.1 mM minimum essential medium nonessential amino acids, 10% minimal essential medium vitamin solution, 0.1 mM bromodeoxyuridine, 100 μM l-ascorbic acid, and 30 mM HEPES, pH 7.2 The cell suspension was subjected to 2 rounds of preplating, each time for one hour to enrich the cardiomyocytes population before plating.

### Isolation of adipose tissue and generation of adipose tissue conditioned medium

For all experiments involving adipose tissue isolation rats were sacrificed by decapitation. For most experiments adipose tissue from adult normal Sprague-Dawley rats was isolated primarily from the epididymal region although some experiments were performed in which the perirenal location was also used, as described in the Results section. Additional experiments were done using epididymal adipose tissue harvested from normal and corpulent (leptin-resistant) JCR:LA-cp rats (University of Alberta, Edmonton, Alberta Canada). After collection, adipose tissue was weighed and minced into small pieces with a surgical blade mounted on a scalpel (~ 50 strokes in criss-cross fashion) in the same culture dish. The tissue was cut with a pair of scissors to ensure that all pieces were cut uniformly and as small as possible and any visible blood capillaries and clots were removed. A 3-dimentional collagen gel consisting of 60% Type 1 collagen was added and mixed to make a homogenous mixture. The suspension was set-aside for half an hour at room temperature to solidify. After solidification, serum free medium was added onto the gel and placed in an incubator at 37°C (5% CO2) for 24 hours. Adipose tissue conditioned medium (ATCM) was then collected for study.

### Experimental protocol

Cardiomyocytes were allowed to grow in serum-containing medium for 24 or 48 hours after which they were washed with PBS and then grown in serum-free medium for 24 hours before treatment. To initiate hypertrophy cells were treated with 10 nM ET-1 (Sigma-Aldrich, Oakville, Ontario, Canada) for 24 hours. The cells were pre-treated for 15 minutes with ATCM before the addition of ET-1 to determine the effect of ATCM. ATCM was diluted from 1.25 x 10^8^ to 20 x 10^8^ to determine the concentration-dependent response. Thus the highest concentration of ATCM used represented a dilution factor of 1.25 x 10^8^ which reflected a titer where complete inhibition of ET-1-induced hypertrophic responses was observed. However, in the case of adipose tissue medium derived from leptin-resistant JCR:LA-cp rats, some experiments were done with undiluted ATCM as shown in Results. Some experiments were also carried out in the presence of an ovine super-active leptin antagonist (LRA, 0.1–10 nM, Protein Laboratories Rehovot Ltd, Israel) or the adiponectin receptor-1 (AdipoR1) antibody (ARA, 1 ng/ml-1μg/ml, Santa Cruz Biotechnology, Dallas, TX). Additional studies were also performed using the 5' adenosine monophosphate-activated protein kinase (AMPK) activator 5-amino-1-β-D-ribofuranosyl-imidazole-4-carboxamide (AICAR, 1 mM, Cell Signaling Technology, Beverly, MA) or the AMPK inhibitor dorsomorphine (Compound C, 0.5 μM-10 μM, Sigma-Aldrich).

### ATCM fractionation using cut-off filters

Millipore Amicon Ultracel-30K 15 ml tubes with 30 kDa filters (Fisher Scientific, Ottawa, Ontario, Canada) were used to fractionate the adipose tissue conditioned medium into two fractions, the lower filtrate containing proteins of less than 30 kDa and the upper concentrate containing proteins greater than 30 kDa. The filter unit was pre-rinsed with nano pure deionized water. The filter unit was filled with 12 ml of ATCM, placed in a 50 ml conical tube and centrifuged at 5000 x g for 30 min at room temperature. At the end of the centrifugation the concentrate was collected from the filter device sample reservoir using a pipette and ultra-filtrate was collected from the provided centrifuge tube.

### Assessment of hypertrophic response

To determine the cell surface area following treatments cell photographs were taken with a Leica inverted microscope equipped with infinity 1 camera (Lumenera Corporation, Ottawa, Ontario, Canada) at 100 x magnification randomly from different areas on the culture plate. Cell surface area was measured using SigmaScan Software (Systat, Richmond, CA) A minimum of 50 randomly selected cells per treatment from each experiment were used to determine surface area and averaged to provide a value of N = 1.

In addition to myocyte surface area we further assessed hypertrophy by determining expression of atrial natriuretic peptide (ANP) and α-skeletal actin. RNA was extracted using TRI reagent (Sigma-Aldrich) according to the manufacturer’s instructions. RNA (1 μg) was used to synthesize the first strand of cDNA using M-MLV reverse transcriptase according to the manufacturer’s protocol and this was used as a template in the PCR reactions. The expression of ANP, α-skeletal actin or TPT1 genes was determined in 10 μl reaction volumes using EvaGreen qPCR Mastermix (Applied Biological Materials, Richmond, British Columbia, Canada). Fluorescence was measured and quantified using a CFX96 Real-Time system (Bio-Rad Laboratories, Mississauga, Ontario, Canada). Amplification was performed using the following primers: 5′-CACGGCATTATCACCAACTG-3′ (forward) and 5′-CCGGAGGCATAGAGAGACA-G-3′ (reverse) for α-skeletal actin, 5′-CTGCTAGACCACCTGGAGGA-3′ (forward) and 5′-AAGCTGT-TGCAGCCTAGTCC-3′ (reverse) for ANP, and 5′- GGAGGGCAAGATGGTCAGTA-3’ (forward) and 5’- AGGCCTCTTTTGTGAAGCTG-3’ (reverse) 3′ (reverse) for tumor protein translationally-controlled 1(TPT1) as the reference gene. PCR conditions to amplify the genes were 30 s at 94°C followed by annealing at the gene-specific temperature (58°C) for 20 s and elongation at 72°C for 30 s. Melting curve analysis showed a single product formation for each gene amplified. We calculated the number of gene copies from a standard curve which consisted of 5 serial dilutions ranging from 1-10^-5^. Each gene expression was normalized using the housekeeping gene TPT1 as an internal control.

### Adipokines analysis in ATCM

Concentrations of adipocyte-derived products in ATCM were analyzed by a commercial laboratory (Eve Technologies, Calgary, Alberta, Canada) using Multiplexing Laser Bead Technology as described in detail on the Company’s web site (https://www.evetechnologies.com/technology.php).

### Western blotting for AMPK, p38, ERK1/2, JNK MAPKs activation

At the end of the 24 hour treatment period protein lysate was prepared and the concentration of protein was determined using the Bio-Rad dye protein assay method (Bio-Rad, Hercules, CA). Fifty micrograms of protein were resolved on a 10% SDS–polyacrylamide gel and transferred to nitrocellulose membranes which were then blocked in 5% milk for 1 h and incubated with primary antibodies, P-AMPK, AMPK (Cell Signaling, 1:1000 dilution), P-p38, P-ERK1/2, P-JNK, p38, ERK1/2, JNK and actin (Santa Cruz, 1:500 dilution) followed by one hour incubation with a secondary antibody conjugated to IRDye (Li-COR, Lincoln, NE). The membranes were then analysed using an Odyssey Clx Infrared Imaging System scanner (Li-COR). The bands were quantified using the Odyssey Application Software (Li-COR).

### Statistical analysis

Results were analyzed using GraphPad Prism 6 software. All data are represented as mean + standard error of the mean (SEM). Data for cell surface area and fetal gene expressions were analyzed using 1-way analysis of variance (ANOVA) followed by a post hoc Tukey test. Data for the concentration response curve of ATCM in the presence or absence of LRA and ARA were analyzed by 2-way ANOVA followed by Bonferroni post hoc test. Results for JCR:LA-cp animals and their lean controls as well as adipokine content in ATCM were analyzed using Student’s unpaired t-test. A value of P < 0.05 was considered significant.

## Results

**Fig 1:**
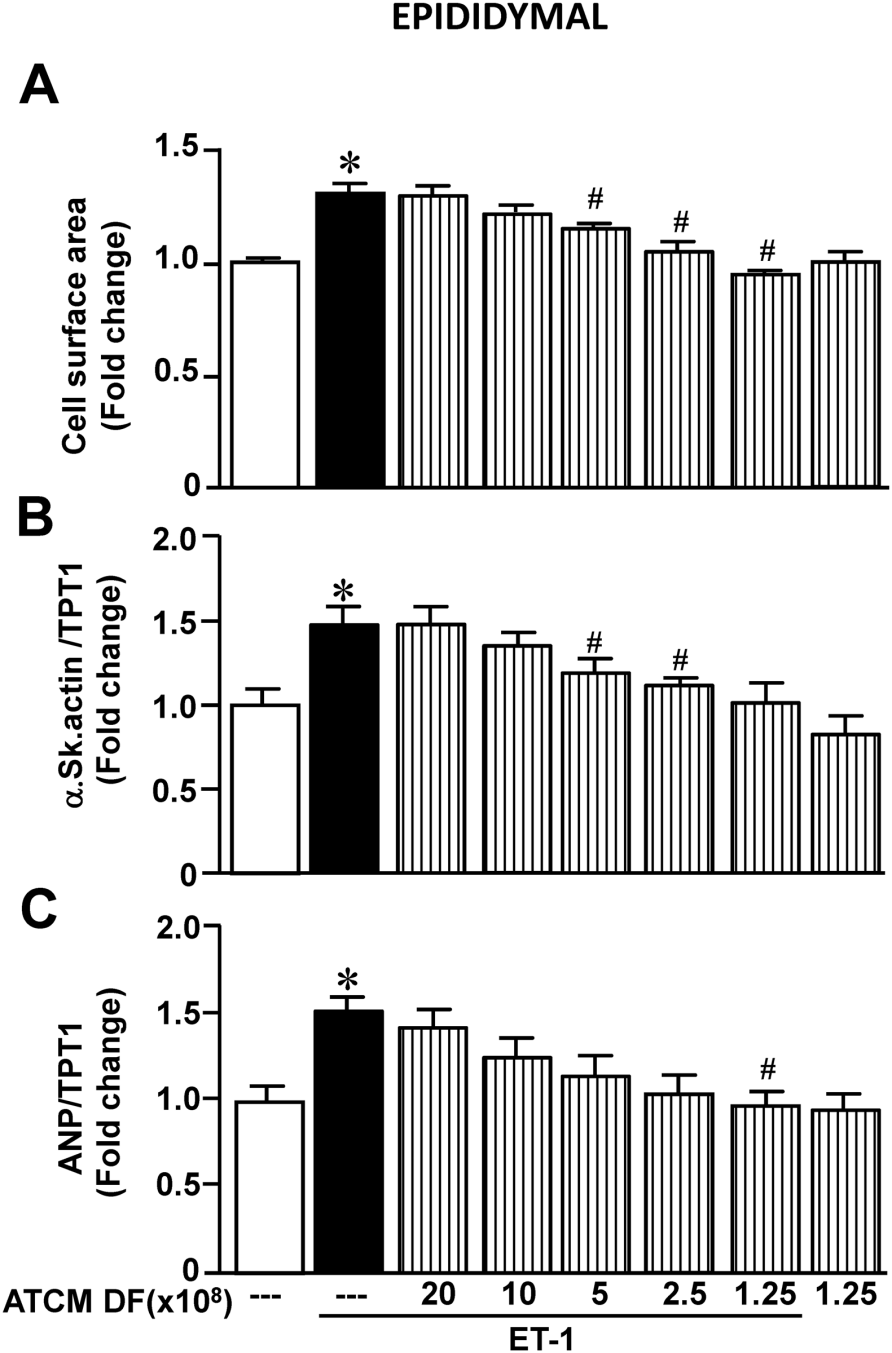
Effect of increasing epididymal adipose conditioned medium(ATCM) concentrations on ET-1-induced hypertrophy as determined by cell surface area (A), expression of α-skeletal actin (B) or expression of ANP (C). Data are expressed as mean + SEM; n = 6–10. *P < .05 from untreated group; ^#^ *P* < .05 from ET-1 alone.

**Fig 2:**
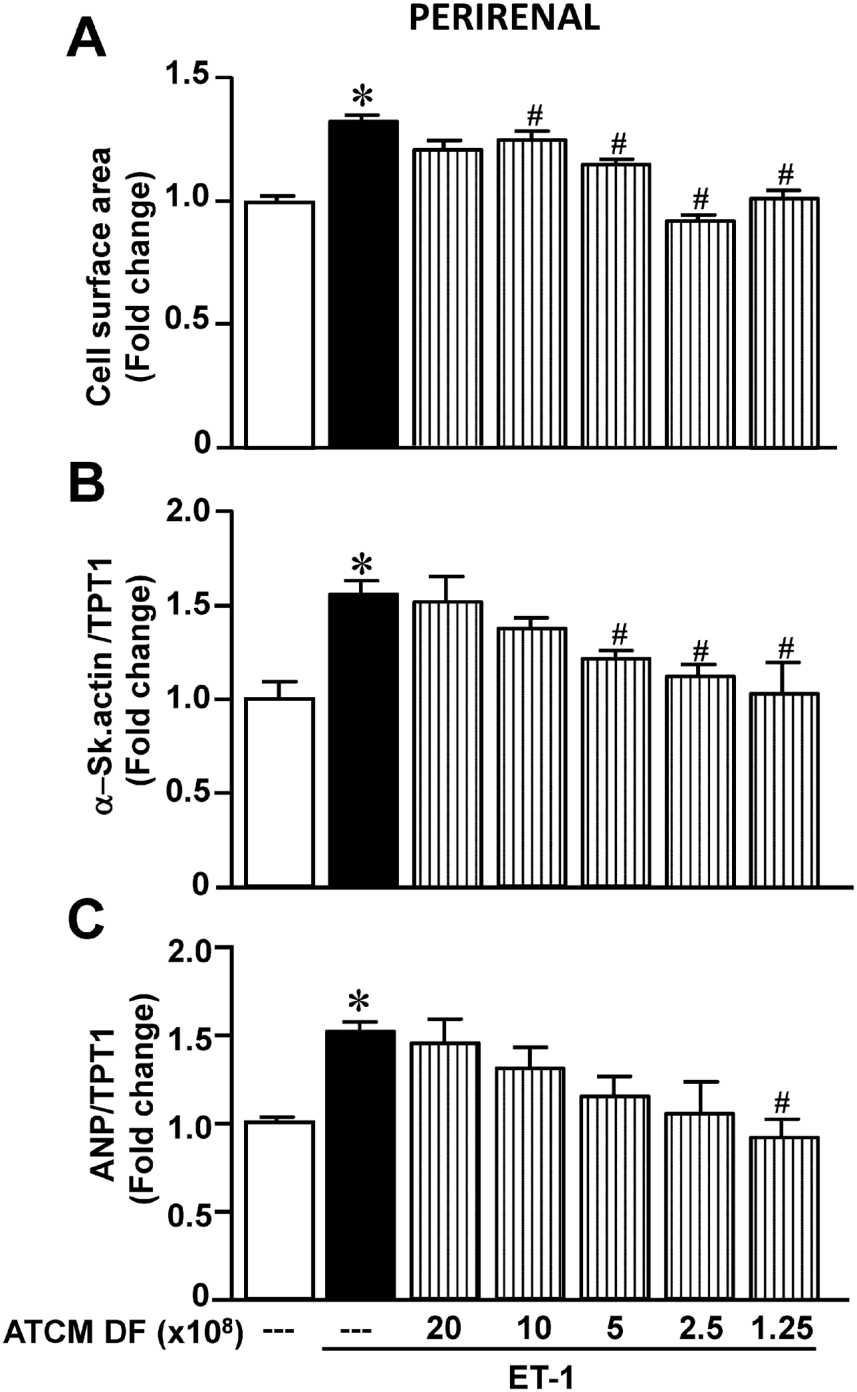
Effect of increasing perirenal adipose conditioned medium (ATCM) concentrations on ET-1-induced hypertrophy as determined by cell surface area (A),expression of α-skeletal actin (B) or expression of ANP (C). Data are expressed as mean + SEM; n = 6–10. *P < .05 from untreated group; ^#^ *P* < .05 from ET-1 alone.

We first determined the ability of epididymal ATCM to modify the hypertrophic effect of ET-1. These results are summarized in Fig 1 and show a concentration-dependent inhibition (based on decreasing dilution factors) of ET-1 induced hypertrophy in terms of cell surface area (panel A), as well expression of α-skeletal actin (panel B) and atrial natriuretic peptide (panel C). The highest ATCM concentration studied (1.25 x 10^8^) completely supressed hypertrophy produced by ET-1 although this concentration of ATCM in the absence of any direct effect on any parameter when administered in the absence of ET-1. We further determined whether ATCM from adipocyte tissue harvested from a different anatomical site, specifically the perirenal region, exerts identical effects. As shown in Fig 2, identical inhibition of the hypertrophic response to ET-1 was evident by ATCM from this adipocyte population similar to that observed with ATCM from epididymal adipocytes. All subsequent experiments were therefore carried out with adipose tissue from the epididymal region.

**Fig 3:**
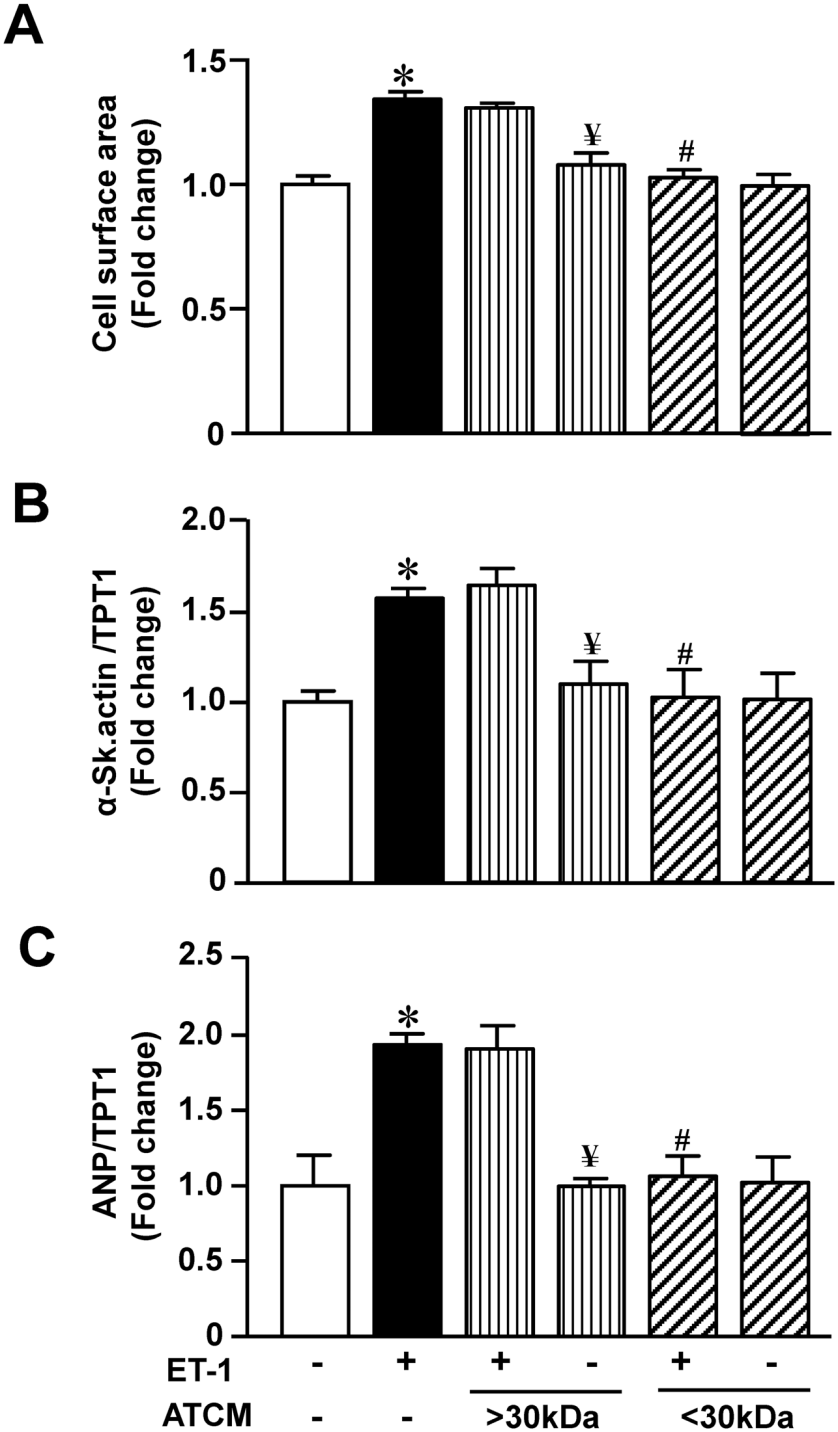
Effect of fractionated ATCM using 30 kDa cut-off filters. Panel A shows surface area for myocytes treated with ET-1 with or without high molecular weight (>30 kDa) or low molecular weight (<30 kDa) ATCM for 24 hours after ET-1 addition whereas B and C show fetal gene α-Skeletal actin and ANP expression, respectively with identical treatments. Values indicate mean + SEM (n = 6). *P < .05 from untreated group; ^*#*^*P<*0.05 from ET-1 alone; ^*¥*^*P<*0.05 from ET-1 + >30kDa ATCM.

**Fig 4:**
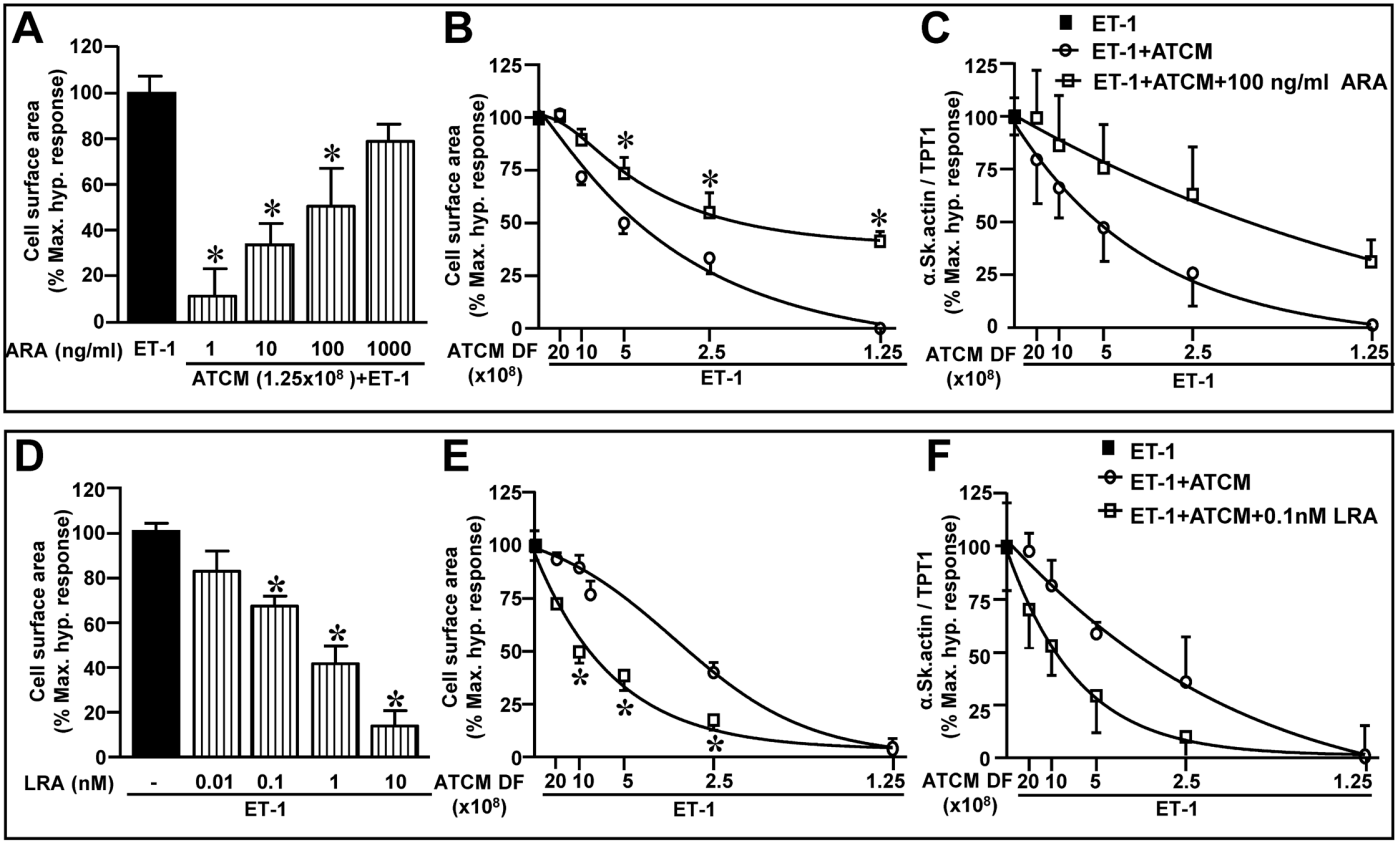
Influence of a adiponectin receptor antibody (ARA) or a leptin receptor antagonist (LRA) on hypertrophic responses. Panel A show the effect of increasing ARA concentrations on ET-1 induced increase in cell surface area whereas B and C show the concentration-dependent effect of ATCM in the presence or absence of 100 ng/ml ARA on cell surface area and expression of α-Skeletal actin, respectively. Panel D summarizes the influence of LRA on cell surface area whereas E and F show the concentration-dependent effect of ATCM in the presence or absence of 0.1 nM LRA on cell surface area and expression of α-Skeletal actin, respectively. Values indicate mean + SEM (n = 6). *P < .05 either from ET-1 alone (A, D) or respective value with ET-1 + ATCM alone (B,E). DF, dilution factor.

The next set of studies was performed to understand the nature of the factor(s) which could potentially mediate the antihypertrophic effect of ATCM. Accordingly as a first step we determined the effect of fractionating adipokine factors using molecular weight cut-off filters to assess the nature of the antihypertrophic efficacy of ATCM. These results are summarized in Fig 3 and show that the antihypertrophic effect of ATCM was maintained in the <30 kDa fraction but completely absent at >30 kDa. A potential basis for these results was that the antihypertrophic effect of ATCM was mediated by adiponectin in view of the established antihypertrophic effect of this protein (see Discussion). We addressed this question in two ways. First, we determined the ability of ATCM to attenuate the hypertrophic effect of ET-1 in the presence of an antibody directed against the AdipoR1 receptor. This antibody was completely devoid of any direct effects on any parameter in the absence of ET-1 (not shown). As shown in Fig 4A, increasing concentrations of the antibody prevented the antihypertrophic effect of ATCM when the latter was used at a dilution factor of 1.25 x 10^8^.

We next determined the ability of decreasing dilutions of ATCM to inhibit the hypertrophic response to ET-1 in the absence or presence of the AdipoR1 antibody at a concentration of 100 ng/ml. These results are shown in Fig 4B and 4C for cell surface area and α-skeletal actin expression, respectively. The results revealed a marked rightward shift in the response to decreasing ATCM dilution in the presence of the antibody. Moreover, at the lowest dilution level complete inhibition of ET-1-induced hypertrophy was absent in the presence of the antibody, in contrast to results seen with ATCM alone (Fig 4B and 4C).

The above results suggest that adiponectin produced by adipocytes represents an important contributor to anti-hypertrophic effect of ATCM. There is emerging evidence that the biological properties of adiponectin may be influenced to an important degree by the presence of leptin, and that the adiponectin to leptin ratio is an important determinant of the net biological effect. To complement the preceding experiment we next determined the effect of leptin receptor blockade on both the myocyte response to ET-1 as well as the anti-hypertrophic effect of ATCM. As shown in Fig 4D, increasing concentrations of the leptin receptor antagonist, which was without any direct effect on its own (not shown) produced a significant inhibition of the hypertrophic response to ET-1, thus mimicking the effect the ATCM. As was done for the AdipoR1 antibody, we next determined the influence of the leptin receptor antagonist on the antihypertrophic effect of decreasing ATCM dilutions. The results, shown in Fig 4E and 4F, reveal a marked leftward shift in the response to ATCM thereby demonstrating increased potency of ATCM to inhibit ET-1-induced hypertrophy under leptin receptor blockade.

**Fig 5:**
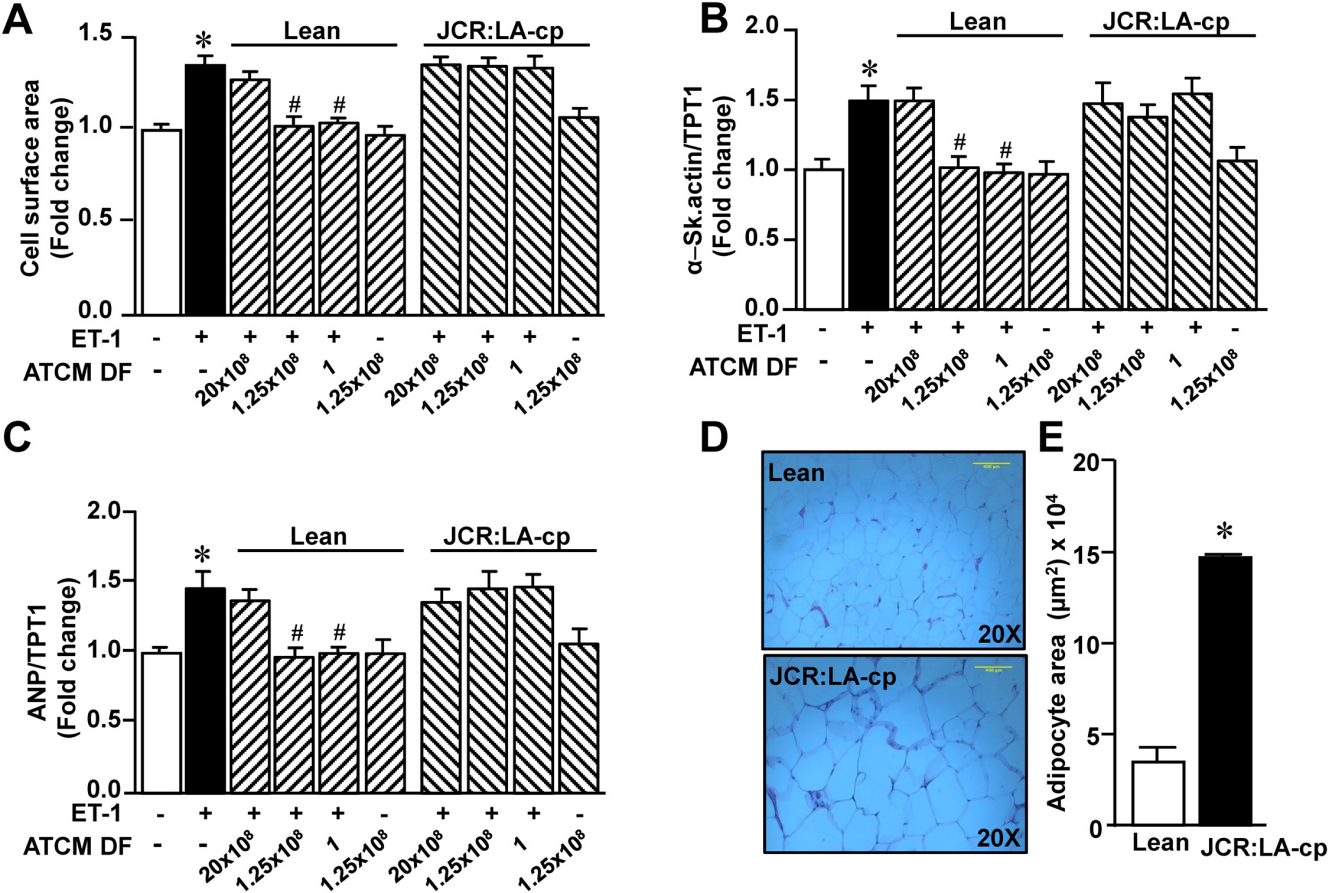
Comparison between the ability of ATCM derived from adipocytes from lean rats versus ATCM from corpulent JCR:LA-cp rats to inhibit the hypertrophic effect of ET-1. Panels A to C show hypertrophic responses to ET-1 under various experimental conditions in terms of cell surface area (A), α-Skeletal actin (B) and ANP (C) expression. Note the inability of ATCM from JCR:LA-cp rats to produce any inhibition of ET-1-induced hypertrophy even with the lowest ATCM dilution. Panels D and E show representative micrographs of adipocytes from the two animal groups and corresponding values for adipocyte area, respectively. Values indicate mean + SEM (n = 6). *P < .05 from untreated group; ^#^*P <* .05 from ET-1 alone group. DF, dilution factor.

To further understand the nature of the antihypertrophic effect of ATCM we determined whether this property of ATCM is altered by obesity. To assess this, we studied ATCM from the JCR:LA-cp rat which exhibits obesity and concomitant leptin resistance and compared the results to lean, metabolically normal controls. JCR:LA-cp animals exhibited a 50% increase in body weight compared to lean controls (510 ± 10 g *vs* 341 ± 7 g, P<0.0001, N = 6). As summarized in (Fig 5A–5C) ATCM from adipocytes of lean rats demonstrated anti-hypertrophic effects against ET-1 similarly to that observed using normal Sprague-Dawley animals. However, as shown in (Fig 5A–5C), this effect was completely abolished when using adipocytes from JCR:LA-cp rats. Fig 5 also demonstrates (panel D and E), that adipocytes harvested from JCR:LA-cp rats were substantially hypertrophied demonstrating a three-fold increase in myocyte area.

**Fig 6:**
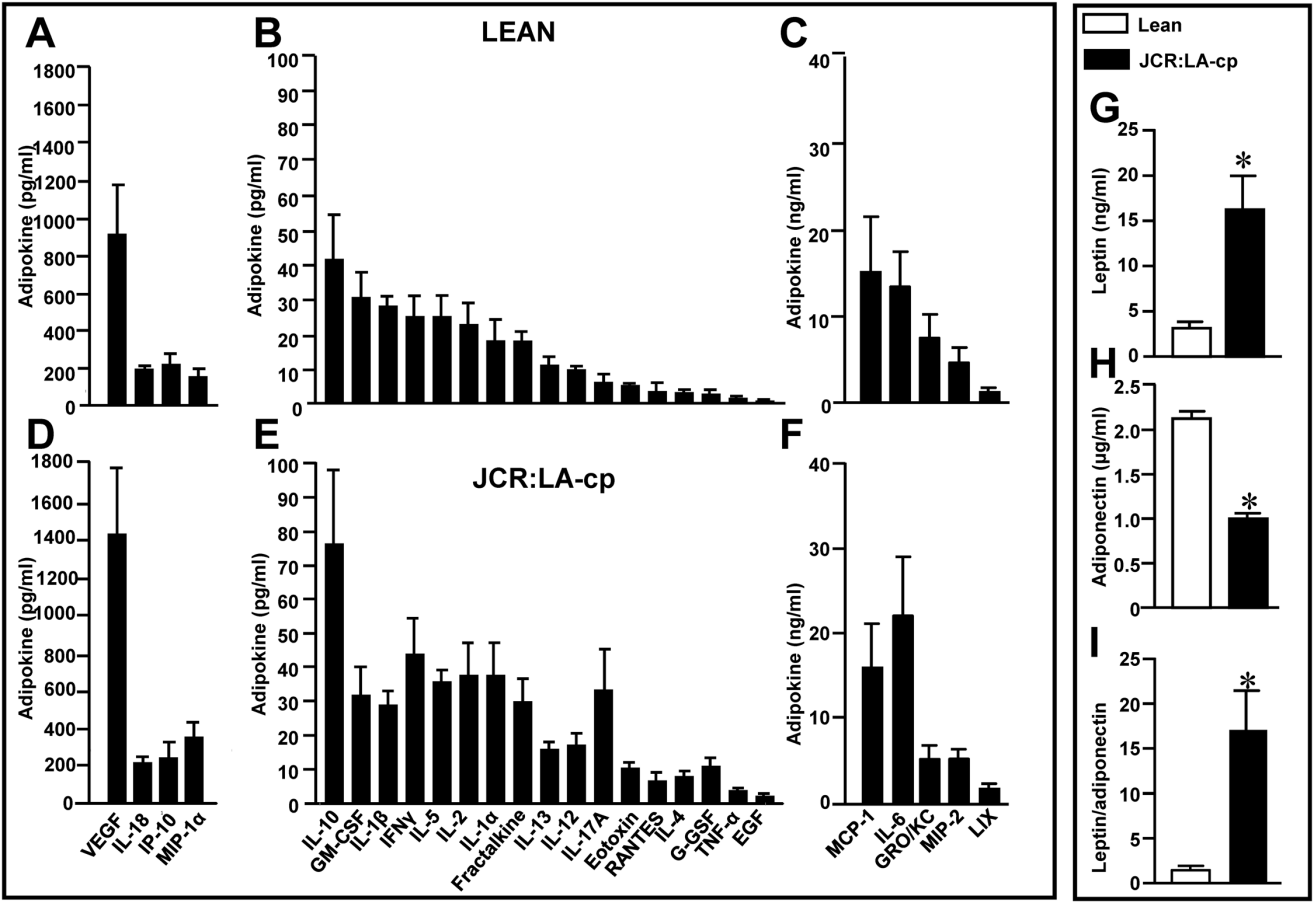
Adipokine content in ATCM derived from adipocytes from lean and JCR:LA-cp rats. Data in panels A to F are grouped based on concentration range whereas values for leptin (G), adiponectin (H) and the leptin to adiponectin ratios (I) for both animal groups are shown separately. Values indicate mean + SEM (n = 6). *P < .05 from lean group.

In order to potentially identify the basis for the lack of effect of ATCM from JCR rats to inhibit ET-1-induced cardiomyocyte hypertrophy we analyzed adipokine content in the two populations. Twenty-seven adipokines were assayed and as shown in (Fig 6A–6F), virtually identical profiles were found for the majority of these products from both animal groups. However, ATCM from JCR rats exhibited a marked significant increase in leptin concentrations (Fig 6G) concomitant with a significant decrease in adiponectin content (Fig 6H) thereby producing a large increase in the leptin to adiponectin ratio (Fig 6I).

**Fig 7:**
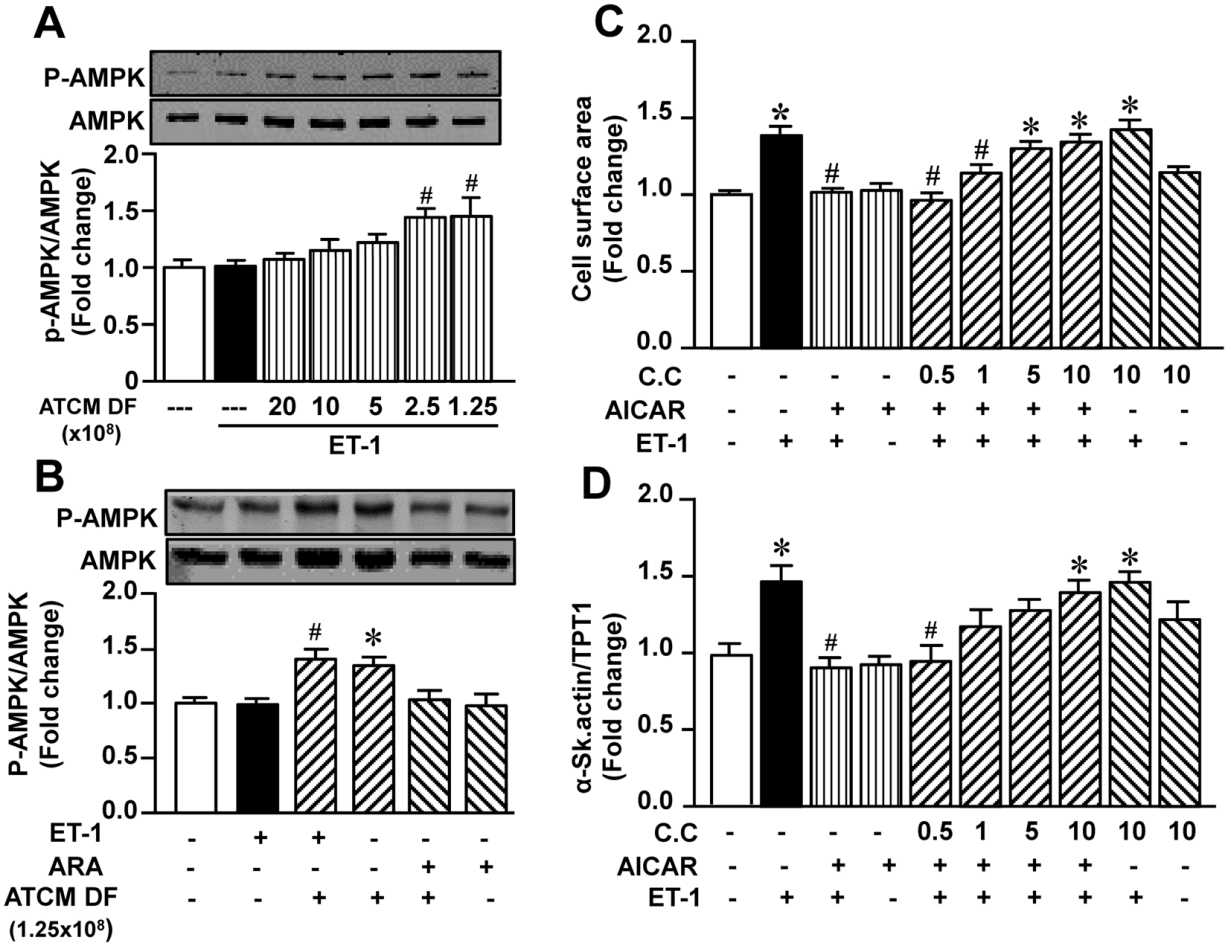
Possible role of AMPK activation in mediating the antihypertrophic effect of ATCM. Panel A shows no effect of ET-1 alone on AMPK phosphorylation as determined by western blotting although the two lowest ATCM dilution levels significantly increased P-AMPK protein expression. The ability of ATCM to increase levels of P-AMPK was prevented by the AdipoR1 receptor antibody (ARA, B). Panels C and D show that the antihypertrophic effect of ATCM can be mimicked by the AMPK activator AICAR, an effect prevented by the presence of the AMPK inhibitor compound C (C.C) in terms of cell surface area (C) and α-Skeletal actin expression (D). Values indicate mean + SEM (n = 6). *P < .05 from untreated group; ^#^*P <* .05 from ET-1 alone group. DF, dilution factor.

The final series of experiments was performed to assess the cellular mechanism underlying the antihypertrophic effect of ATCM. As our results suggest that the primary mediator for this effect is adiponectin and as a major target of adiponectin is AMPK phosphorylation/activation we focussed primarily on the regulation of AMPK activity and influence of ATCM in presence or absence of AdipoR1 antibody on cardiomyocytes treated with ET-1. Moreover, we determined whether the antihypertrophic effect of ATCM can be mimicked by the AMPK activator AICAR. As Fig 7A illustrates, addition of ATCM produced a concentration-dependent increase in AMPK phosphorylation, which reached significant values at the two lowest dilution factors. Although ET-1 alone had no effect on AMPK phosphorylation, a significant increase was seen in the presence of ATCM at a dilution of 1.25 x 10^8^. This effect was due solely to the presence of ATCM as the increase in AMPK phosphorylation was identical irrespective of the presence or absence of ET-1 (Fig 7B). Importantly, the effect of ATCM on AMPK phosphorylation was completely abrogated by the AdipoR1 antibody, although the latter was without effect on its own (Fig 7B). Furthermore, as shown in Fig 7C and 7D, the effect of ATCM on ET-1-induced hypertrophy was mimicked by AICAR, an effect completely prevented by increasing concentrations of compound C, a potent AMPK inhibitor.

**Fig 8:**
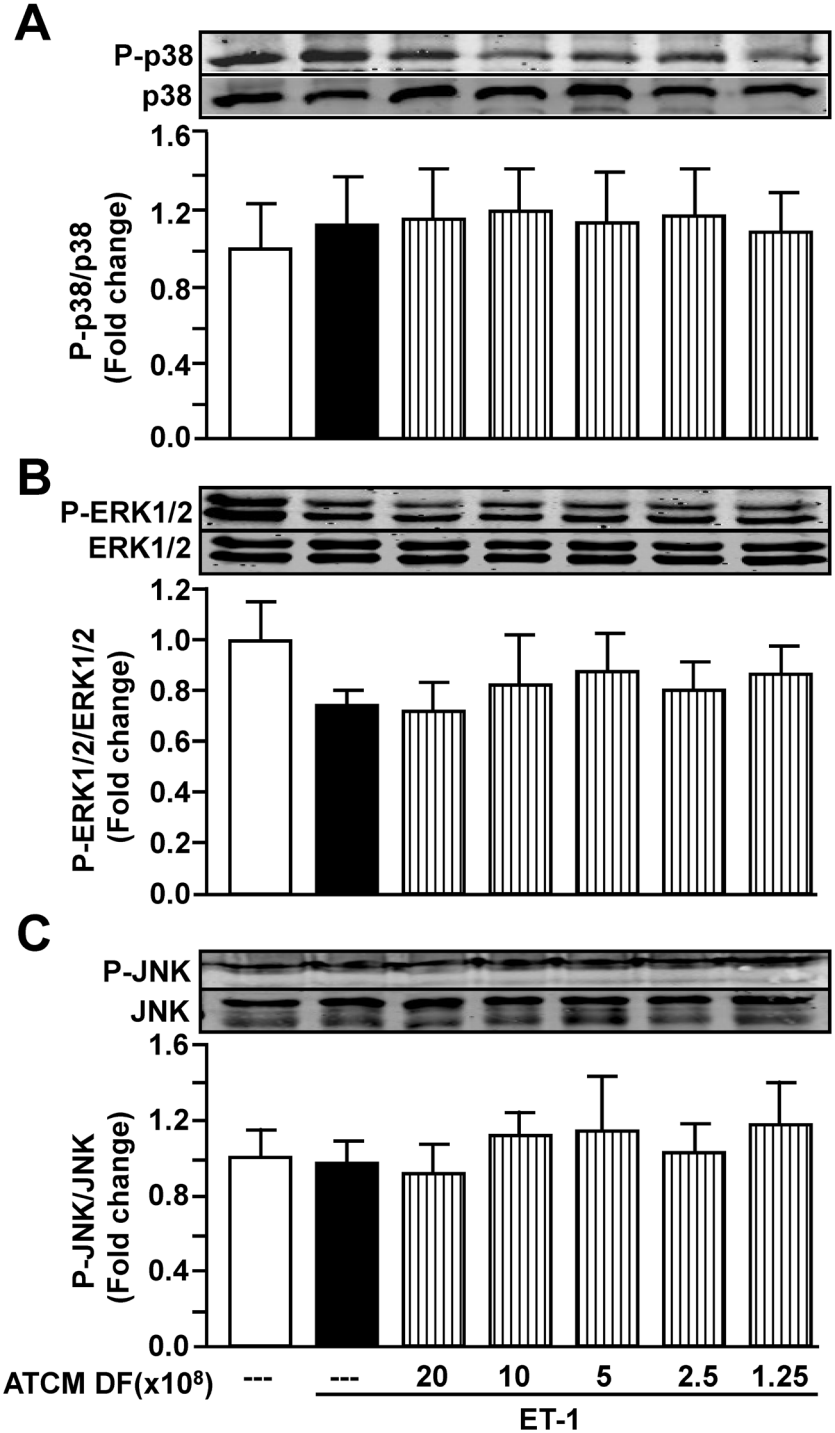
Total and phosphorylated MAPK activities under various experimental conditions. Panels A, B and C show representative western blots and the corresponding quantified values for p38, ERK1/2 and JNK activation, respectively. Values indicate mean + SEM (n = 6). DF, dilution factor.

MAPKs are also considered as potential mediators of cardiac hypertrophy and, accordingly, we determined the effect of ET-1 on p38, ERK/12 and JNK activation in the absence or presence of ATCM. However, as shown in Fig 8A–8C, no effect on MAPK activity as assessed by phosphorylation status was found under any experimental condition 24 hours after ET-1 administration.

## Discussion

Increased adiposity is generally considered as a contributing factor to the development of disease states particularly with respect to cardiovascular disorders with secretion of bioactive adipokines considered as potential contributors to this process [5, 19]. Two adipokines which have received extensive attention over the past number of years are the 16 kDa satiety factor leptin [20] as well as adiponectin which exist in plasma as three oligomeric units [21]. Substantial evidence during the past number of years has suggested that an unfavourable leptin to adiponectin ratio, that is high plasma leptin vs low adiponectin plasma concentrations may predispose individuals to increased cardiovascular risk [6–9].

Although many adipokines exert effects on the cardiovascular system [22] little is known as to whether adipocytes, or more specifically, ATCM can affect cardiovascular responses. The use of culture or co-culture models offer a very useful approach for the study of myocyte-adipocyte interaction and to determine how adipocytes could affect cardiomyocyte behaviour [23]. Using conditioned medium from human adipocytes it has been previously shown that these adipocytes release a cardio-depressant factor, identified as the adipocyte fatty acid binding protein FABP4 that acutely produces a direct inotropic effect, a property which may contribute to cardiac dysfunction associated with obesity [17]. Our objective in the present study was to determine whether ATCM can modulate more chronic effects on cardiomyocytes and in this regard we sought to determine whether administration of ATCM can alter the hypertrophic response of cardiomyocytes to ET-1 and if so, to attempt to identify the nature of the effect as well as potential underlying mechanisms. We show for the first time that ATCM obtained from adipose tisssue from lean rats can completely supress the hypertrophic response to ET-1 whereas, as discussed below, this effect is lost when using ATCM from obese animals. We also show that the ability of ATCM to inhibit the hypertrophic response was independent of adipocyte anatomic location as identical effects were seen with adipose tissue harvested from either epididymal or perirenal locations, the most readily accessible sites for adipocyte collection on normal rats. Our findings strongly implicate a role for adiponectin in the anti-hypertrophic influence of ATCM and suggest that the balance between leptin and adiponectin production represents the primary factor dictating the net effect of ATCM. This is supported by multiple lines of evidence including the ability of a highly specific leptin receptor antagonist to enhance the anti-hypertrophic effect of ATCM whereas an antibody directed against the AdipoR1 receptor attenuated the antihypertrophic influence of ATCM. Moreover, as discussed below, the differences in effects observed using adipose tissue conditioned media from obese versus lean rats appears to be dictated primarily by relative levels of leptin and adiponectin found in the ATCM since no differences were observed in levels in any of the other 25 adipokines measured.

Leptin exerts its effect primarily by activating the long form of its receptor termed OBRb (or LEPRb) which is expressed in the cardiomyocyte and which results in activation of the prohypertrophic program [24, 25]. In contrast, adiponectin is an anti-hypertrophic factor which exerts its effect by activating specific receptors also expressed in cardiomyocytes termed AdipoR which are then subdivided into either the AdipoR1 or AdipoR2 subtypes [26, 27]. Although there is evidence that silencing AdipoR2 supresses the anti-cardiac hypertrophic effect of adiponectin [28], the primary response to adiponectin reflects AdipoR1 in heart [27]. We used multifaceted approaches to identify the nature of the anti-hypertrophic effect of ATCM and the underlying mechanistic bases. To identify the general nature of potential mediators for the anti-hypertrophic effect of ATCM, we used different molecular weight cut-off filters and demonstrated that the factors(s) mediating the anti-hypertrophic effect had molecular weights of between 3 and 30 kDa, a finding applicable to the full-length adiponectin protein or globular adiponectin although as noted above adiponectin can also circulate as trimer, hexamer and high molecular weight oligomer isoforms [21]. These different adiponectin isoforms also possess distinct abilities to stimulate cell signalling processes [29]. The use of a highly specific OBRb antagonist [30] revealed a marked sensitization of myocytes to the anti-hypertrophic effect of ATCM, suggesting that the presence of leptin mitigated the ATCM-induced anti-hypertrophic effect. Conversely, an antibody directed against AdipoR1 significantly attenuated the ability of ATCM to reduce the pro-hypertrophic effect of ET-1 thus implicating adiponectin as a mediator of this effect. There is now compelling evidence that many of the beneficial effects of adiponectin including its role as an antihypertrophic factor is related to AMPK activation [31–34] although how AMPK exerts salutary effects is not known with certainty. The ability of ATCM to increase AMPK phosphorylation is suggestive of an important role of the latter in mediating the antihypertrophic effect of ATCM through an adiponectin-dependent pathway. This was reinforced by the fact that the AdipoR1 antibody abrogated ATCM-induced AMPK phosphorylation. It is also interesting that AICAR, an AMPK activator [35] was able to mimic the inhibitory effect of ATCM on ET-1 induced cardiomycyte hypertrophy, an effect abrogated by compound C, an AMPK inhibitor [36].

We also determined the potential role of the MAPK family of enzymes in terms of mediating the hypertrophic effect of ET-1. Surprisingly, we observed no effect on MAPK activation by ET-1 at least 24 hours after addition of the peptide, suggesting that an earlier time point measurement could have revealed MAPK activation. It should be noted that in addition to MAPK many other pathways and mediators have been identified as potential contributors to ET-1-induced cardiomyocyte hypertrophy [37–40] such that a precise mechanism or mechanisms by which ATCM supresses ET-1-induced hypertrophy requires substantial further studies.

**Fig 9:**
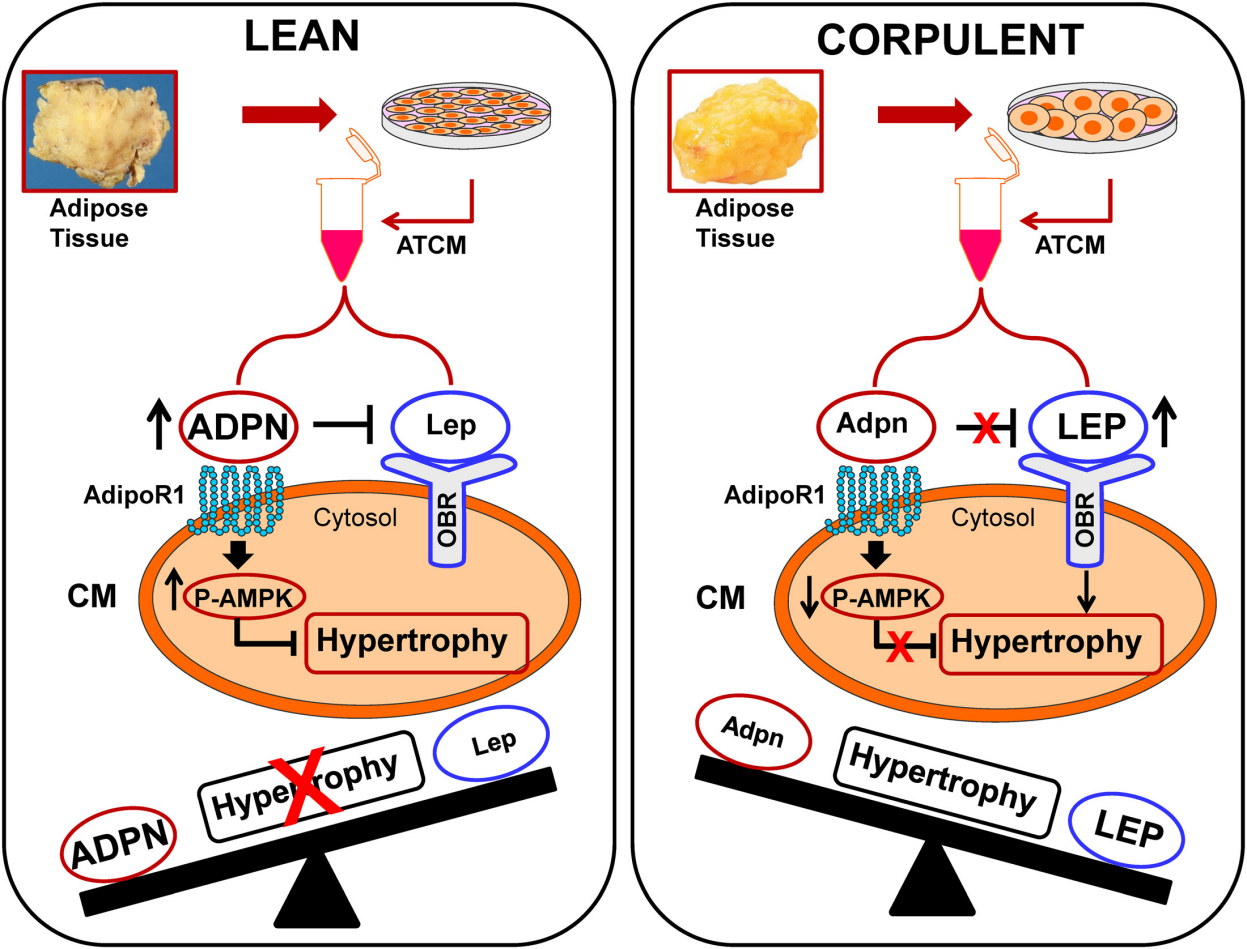
Postulated mechanisms underlying opposite effects of ATCM from lean and corpulent animals on the hypertrophic response in cardiomyocytes (CM). ATCM from lean animals secrete copious quantities of adiponectin (ADPN) which activates the AdipoR1 receptor and blocks the hypertrophic response, possibly via increased AMPK phosphorylation (P-AMPK). This condition favours the production of adiponectin in relation to leptin (Lep) which also allows adiponectin to block the prohypertrophic effect of leptin. Although the concentration of adiponectin in ATCM is substantially higher under all conditions when compared to leptin, the ratio of leptin to adiponectin increases dramatically under obesity as reflected by increased leptin release (LEP) concomitant with decreased adiponectin (Adpn). This change in the adiponectin to leptin relationship results in a diminished antihypertrophic effect of adiponectin since its effects can be countered by leptin. At the same time the increased levels of leptin, coupled with decreased adiponectin allows potentially for a direct prohypertrophic effect of leptin acting via its receptor (OBR). As illustrated at the bottom of each panel, the ratio of leptin to adiponectin will dictate the overall hypertrophic response, even though the absolute levels of adiponectin remain substantially higher.

To study the potential influence of obesity on responses to ATCM we obtained adipose tissue from JCR:LA-cp rats which is one of a number of strains that incorporate the autosomal recessive *cp* gene. Rats that are homozygous for the gene (*cp/cp*) develop obesity, insulin resistance, hypertriglyceremia and various other abnormalities thus providing a suitable model of metabolic syndrome which is similar to humans [41, 42]. Intriguingly, ATCM from adipocytes harvested from these animals failed to inhibit ET-1 induced cardiomyocyte hypertrophy whereas the anti-hypertrophic effect was maintained in JCR lean animals. We attempted to identify the basis for the different responses by performing an extensive analysis of adipokines present in ATCM from both groups of animals. These results strongly support the notion that the inability to produce the anti-hypertrophic response in ATCM from JCR:LA-corpulent rats likely reflected the increased leptin levels in ATCM from these animals coupled with decreased adiponectin levels resulting in an eleven-fold increase in the leptin to adiponectin ratio. Quite remarkably, the obesity-induced changes were extremely selective in that the levels of all other adipokines were not significantly changed. Direct evidence for white adipocyte remodelling in obesity has recently been presented in a study showing that high fat diet-induced obesity produces substantial alterations in adipocyte metabolic profile and defects in mitochondrial structure and function [43]. Our findings show a distinct aspect of obesity-induced adipocyte remodelling exemplified by reduced adiponectin and enhanced leptin secretion. A postulated conceptual framework to explain the differences between ATCM from lean and corpulent animals is illustrated in Fig 9.

There is now substantial evidence from both animal and clinical studies that the leptin to adiponectin ratio represents a critical and likely a more accurate index for various cardiovascular- and metabolic-related morbidities compared to each component alone [6–9]. However, a potential role for adiponectin as a risk factor for the development of heart failure has also been suggested [44]. Our study suggests that obesity can produce adipocyte remodelling by virtue of the cell’s ability to increase leptin production concomitant with a marked reduction in production of adiponectin although extensive further studies are necessary to understand the trigger factor for initiating such remodelling as well as the mechanisms underlying this effect.

When taken together the results of the present study suggest that adipocytes in non-obese subjects may function as salutary anti-hypertrophic factors, a property reflecting a favourable leptin to adiponectin balance, which is compromised in obesity due to adipocyte remodelling. An important area to address is whether epicardial adipose tissue which is considered as a risk factor for various cardiac pathologies but which can also exert salutary effects under some conditions [45–47] can also produce such varied effects and whether these effects can similarly be modulated by the presence of obesity. Moreover, there is evidence that conditioned medium from epicardial adipose tissue derived from patients with type 2 diabetes mellitus induces dysfunction of rat ventricular myocytes *ex vivo* whereas conditioned medium from adipose tissue biopsies from non-diabetic patients was without effect [48]. Although modulation of hypertrophic responses was not determined in that study, nonetheless it reinforces the concept that underlying pathology can greatly determine adipocyte function. Taken together, it thus appears that epicardial adipose tissue can produce both salutary and deleterious effects, the nature of which being dependent on clinical state, although whether these differences can also apply to hypertrophic responses is not known. In view of the paucity of epicardial adipose tissue present in the rat, this study will need to be addressed using other experimental paradigms.
